# High migratory propensity constitutes a single stock of an exploited cutlassfish species in the Northwest Pacific: A microsatellite approach

**DOI:** 10.1371/journal.pone.0265548

**Published:** 2022-03-17

**Authors:** Yu-Hong Guo, Lorenzo C. Halasan, Hui-Yu Wang, Hsiu-Chin Lin

**Affiliations:** 1 Department of Marine Biotechnology and Resources, National Sun Yat-sen University, Kaohsiung, Taiwan; 2 Institute of Oceanography, National Taiwan University, Taipei, Taiwan; 3 Doctoral Degree Program in Marine Biotechnology, National Sun Yat-sen University, Kaohsiung, Taiwan; National Cheng Kung University, TAIWAN

## Abstract

Cutlassfishes, also known as hairtails, include multiple predatory fishes of the family Trichiuridae. They constitute a top marine fish commodity globally, yet the knowledge about their composition and intraspecific genetic structures is still limited. *Trichiurus japonicus* accounts for a major amount in the northwest Pacific fishery. Previous studies based on mitochondrial DNA markers reported incongruences in its population structure, hence prompting the need for high-resolution markers and avoiding possible shortcomings in its management. Here we genotyped ten novel *de novo*-assembled transcriptome-derived microsatellite markers on a total of 150 samples across five major fishing grounds (encompassing latitudes 22–39°N). These markers presented a high number of alleles and heterozygosity compared to other marine fishes, corresponding to the large effective population size of ~20,000 per location and cohort differentiation. Population structuring analyses suggested *T*. *japonicus* to be a homogenous well-mixed population. This configuration is likely attributed to the majority of its effective population migrates across locations, and the absence of oceanographic barriers at the continental shelves. Qingdao with reportedly high ocean productivity could be a genetic pseudosink based on the high heterozygosity and migratory preference. Moreover, the results of sign tests suggest that *T*. *japonicus* experienced a recent bottleneck likely concurrent with historical glaciation events. Further, we demonstrated satisfactory cross-amplifications of our markers on several congeners, indicating a great promise to use these markers to study the population genetics of trichiurids. Together, our findings will serve as an essential groundwork for enhancing resource conservation and management of cutlassfishes.

## Introduction

Cutlassfishes or hairtails are predatory fish species of the family Trichiuridae. They are characterized by extremely elongated and compressed bodies and inhabit the tropical and temperate continental shelves [1, 2]. Trichiurids possess high commercial value, which is illustrated by their annual >1.2 million tons catch volume [3]. About 85% of its capture fishery originated from the Northwest (NW) Pacific. In this region, the dominant and most harvested cutlassfish species is *Trichiurus japonicus* [4–7].

Intra-specific variations were observed in the NW Pacific-endemic *T*. *japonicus*. Life-history traits such as growth rate, maturation time, and maximum body length, as well as an inverse correlation to temperature, were found variable [8]. Besides its year-round spawning ability, two spawning peaks observed from the Taiwan coast in February-July and November-December demonstrated the capability of *T*. *japonicus* for multiple cohort productions per year [9]. In addition, movements across spawning and feeding grounds, with a close association with the gonadal development, were also detected from the East China Sea observations [10]. These variations were somehow expected for a marine species with a wide latitudinal range (22–39°N). For similar reasons, this also stimulated interests on whether NW Pacific populations constitute a single genetic unit or an integration of genetically different clusters.

Environmental factors such as oceanographic features and geological barriers are known to differentiate populations in various marine organisms [11]. In the NW Pacific, several ocean currents with unique features (e.g. temperature, salinity, and directional flow) interplayed with each other and configured heterogeneous environments, both spatially and temporally. This interplay of current systems is mainly influenced by the Yellow Sea Warm Current (YSWC) [12], the Yellow Sea Cold Water Mass (YSCWM) [13, 14], Taiwan Strait Water Mass (TSW) [15], East China Sea Shelf Surface Water (ECSSSW), and the Kuroshio Branch Currents (KBC) [16]. Furthermore, the massive freshwater discharge of the Yangtze River, also known as the Changjiang Diluted Water (CDW) [17], is also recognized as an oceanographic barrier for some nearby marine organisms [18, 19]. In Tzeng et al. (2016), the partitioning between East and South China Seas populations was attributed to the hindered gene flow of *T*. *japonicus* populations, which was caused by the exposure of Taiwan Strait during the glacial periods [20].

Genetic tools have proven to be efficient in identifying and characterizing the presence of different populations or the so-called stocks in fisheries [21–23]. These populations react to natural and anthropogenic impacts differently and thus should be treated as separate units for conservation and sustainable management [24]. Population genetics of *T*. *japonicus* have only been studied using mitochondrial DNA markers, including cytochrome b (Cyt-*b*) [20, 25], control region (D-loop) [26], and 16S rRNA [6] gene regions. Although there is a general pattern of homogeneity and lack of intra-specific differentiation, only one study based on Cyt-*b* and control region revealed two genetically differentiated populations at the East and South China Seas, with Taiwan Strait as their transient zone [20]. These incongruent results display the limitation of mitochondrial DNA markers in assessing intra-specific patterns for *T*. *japonicus*. Other genetic markers, which are non-mitochondrial in origin and have a higher capacity to yield better resolutions, would help elucidate this unresolved intra-specific genetic pattern.

Microsatellites or simple sequence repeats (SSRs) are nuclear DNA with short and tandemly repeated simple sequences. SSRs have been widely used as markers for population genetics due to their high polymorphicity [27]. Their high mutation rate makes them highly variable [28–30], thus providing good genetic resolutions for species targeted for conservation and management [31]. Recent developments in the genotyping protocols and software performances also improved the accuracy of applying microsatellite SSRs [32]. There are two types of microsatellite markers based on where the original sequences were derived from—genomic-SSRs (gSSRs) from genomic sequences and expressed sequence tag-SSRs (EST-SSRs) from transcribed RNA sequences. Ever since the development of next-generation sequencing technologies [33], the recovery of transcribed RNA sequences became cost-effective and less labor-intensive through massively parallel sequencing; and this was then applied to screen for EST-SSRs even for non-model organisms [34, 35]. Compared to gSSRs, EST-SSRs have the advantage of more accurate allele binning [36], high association to functional genes [37], transferability across related species [38], and lesser null alleles [39]. To date, EST-SSRs are still popularly used for studying the population genetics of fishery species [40–42], and some studies have successfully elucidated population structures in the NW Pacific [43, 44]. For cutlassfishes, only a few microsatellite loci have been developed and characterized, and they were exclusively limited to *T*. *haumela* [45], *T*. *lepturus* [46], *T*. *nanhaiensis* [47], *Eupleurogrammus muticus* [48], and *Lepturacanthus savala* [49]. Only one study subsequently applied them to conduct population genetic analyses in the Savalai hairtail (*L*. *savala*) along the coast of China [50]. Population genetic studies using microsatellite markers on *T*. *japonicus* from the NW Pacific are still unavailable.

**Fig 1:**
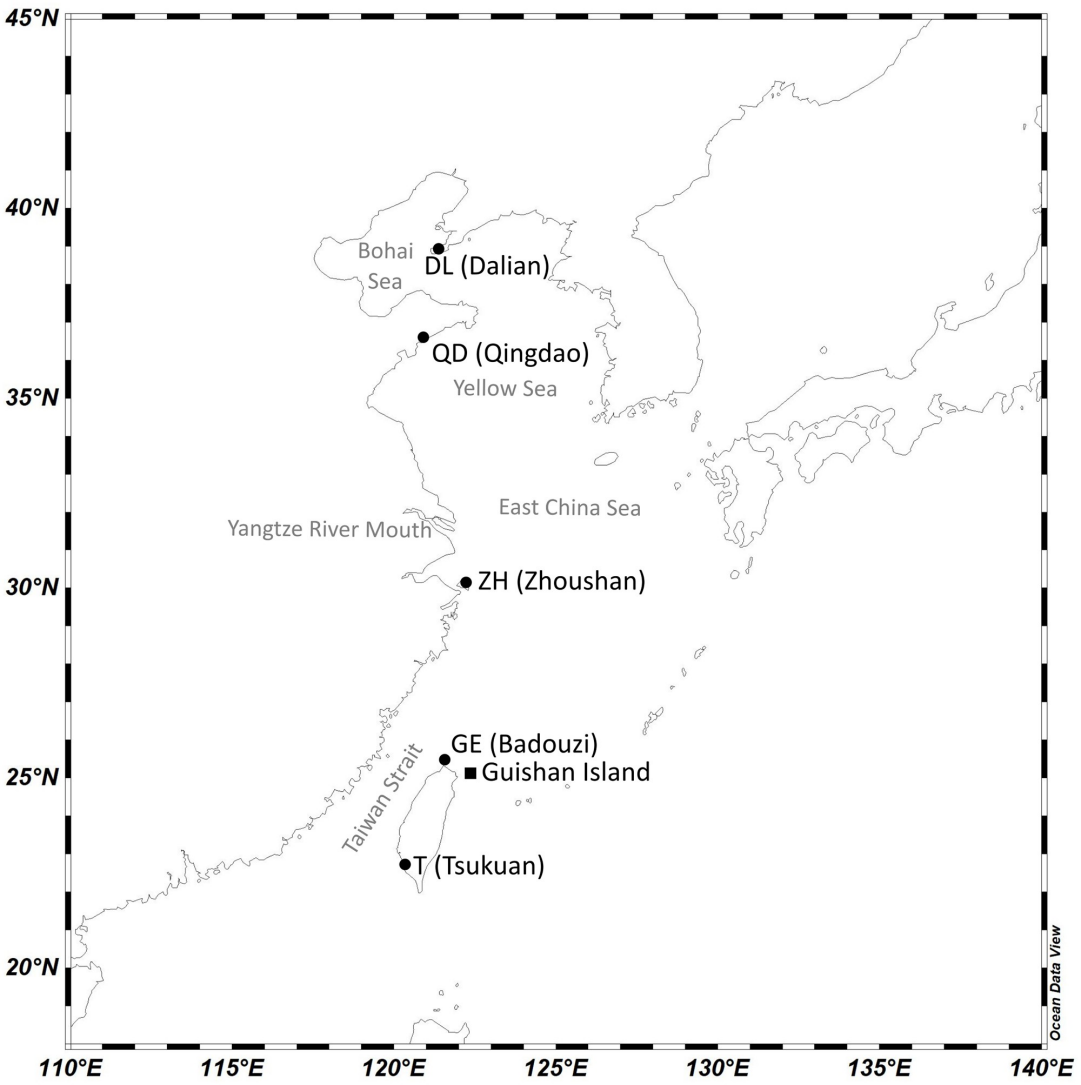
Map of the Northwest Pacific illustrating the sampling locations of *T*. *japonicus* for microsatellite study (circles) and RNA extraction (square). The details of sampling location and time are described in Table 1. The map was generated by Schlitzer, Reiner, Ocean Data View, odv.awi.de, 2021.

This is the first study to use microsatellite markers in determining the population genetics of *T*. *japonicus*. We hypothesized the presence of an isolated population in the Yellow Sea based on its semi-enclosed topology, unique oceanographic dynamics, and the freshwater barrier from the Yangtze River (Fig 1), as well as in Taiwan Strait where historical vicariance events occurred. We performed transcriptome sequencing using Illumina and developed microsatellite markers accordingly. These markers were then used to study the population structure and demographic history of *T*. *japonicus*, which provide relevant information to determine the genetic boundaries for management and formulate sustainable cutlassfish fishery resource strategies.

## Materials and methods

### DNA sample collection and extraction

Thirty adult *T*. *japonicus* individuals were randomly selected from the fisheries catch at each of the five fishing grounds covering the distribution range along the NW Pacific coast, namely: Dalian (DL), Qingdao (QD), Zhoushan (ZH), Badouzi (GE), and Tsukuan (T), from August 2018 to November 2019 (Fig 1, Table 1). A muscle tissue sample from each individual was isolated and preserved in 95% Ethanol. Total DNA used in the microsatellite experiments was extracted from the tissue sample using BioKit Tissue & Cell Genomic DNA Purification Kit (BioKit, Taiwan) following the manufacturer’s protocol.

**Table 1:** Sampling location and time of *T*. *japonicus* specimens collected in this study.

Location	ID	Latitude	Longitude	Time
Dalian	DL	38.912°N	121.602°E	2019 Sep, Oct
Qingdao	QD	36.066°N	120.369°E	2018 Oct; 2019 Nov
Zhoushan	ZH	29.989°N	122.205°E	2018 Oct, Dec; 2019 Jan
Badouzi	GE	25.130°N	121.767°E	2018 Aug
Tsukuan	T	22.725°N	120.252°E	2019 May, Jul, Sep, Oct, Nov
Guishan Island	-	24.801°N	121.978°E	2020 Feb

### RNA sample collection, extraction, and sequencing

One *T*. *japonicus* individual was collected off Guishan Island, Taiwan (Fig 1, Table 1) in February 2020 for RNA extraction. A fresh muscle tissue sample was preserved in Ambion RNAlater solution (ThermoFisher Scientific, USA) prior to the extraction. Total RNA was extracted with RNeasy Mini Kit (Qiagen, Germany) and eluted with 40 μl of RNase-free water from the RNeasy Mini columns. Following the extraction, the purity of the RNA was assessed and quantified using NanoDrop 2000 spectrophotometer (ThermoFisher Scientific, USA). Fragments of the RNA were also assessed in a 1.5% agarose gel electrophoresis. After the quality control procedures, the mRNA was enriched from total RNA using oligo (dT) beads and fragmented randomly in a fragmentation buffer. Random hexamers were then used to reverse-transcribe the mRNA into cDNA. The cDNA library of *T*. *japonicus* sample was prepared by Illumina TruSeq RNA Sample Prep Kits v2 and sequenced using HiSeq 2500 High-Throughput Mode v4 with paired-end 125 basepair reads operated by Novogene Co., Ltd., according to the manufacturer’s instructions (Illumina, San Diego, CA).

### Sequence *de novo* assembly

The sequence read quality was checked and filtered with FastQC v0.11.5 [51] and subjected to Trimmomatic v0.35 [52] for adaptor trimming and rejection of low-quality reads. In obtaining the assembled transcriptome, the clean reads were *de novo* assembled in Trinity v2.2.0 [53, 54]. Corset v1.06 [55] was then used to remove contig redundancies by hierarchical clustering. Afterward, the longest transcripts of each cluster were selected as unigenes and were used for the succeeding microsatellite loci detection.

### Microsatellite locus analysis and primer design

Microsatellite identification tool (MISA) v1.0 was used for microsatellite loci detection in the unigenes [56, 57]. The following minimum number of repeats of each unit size were established as default parameters: mononucleotide repeats ≥ 10; dinucleotide repeats ≥ 6; tri-, tetra-, penta- and hexa-nucleotide repeats ≥ 5. Studies have shown that more information can be drawn out from higher repeat numbers, whereas repeat numbers <10 have already low variability [58, 59]. Perfect repeats were also observed to be more polymorphic than imperfect repeats [60]. Therefore, we did preliminary tests on microsatellite loci with perfect repeats; and the top 22 highest number of repeats, which all have >10 repeats, were utilized as markers for this study. The sequences generated were used to design primers with the Primer3 v2.3.5 program [61] based on the following criteria: (1) primer lengths must be 18 to 23 bp and (2) polymerase chain reaction (PCR) product size must be below 350 bp. Afterward, 22 pairs of primers were manufactured and tested for PCR amplification. Based on initial screenings, 10 pairs ensued successful amplification and thus were chosen as the final microsatellite markers for the succeeding analyses (Table 2). The 5’ end of each forward primer was modified to contain fluorescence (FAM-, HEX-, NED-, and TET-).

**Table 2:** The characteristics of the ten microsatellite loci of *T*. *japonicus* developed from the transcriptome dataset.

Locus	Motif	Primer sequence (5’-3’)	*T*a(°C)
TJ-2	(CA)_43_	F: HEX-CCTTCATCAGAGCACTGCCA	66.5
		R: ACTGACCACACATCACACTGA	
TJ-10	(TG)_29_	F: FAM-AGACATGATGGTGTGTGCGT	64
		R: GAACAGATTCTCCCGCCGAT	
TJ-8	(GT)_32_	F: TET-GTTTCTTTGCAGCAGGCTCA	54.9
		R: ATGTAATCGGCCCCTGAAGC	
TJ-9	(TG)_30_	F: NED-CAAATTAGCCCCACAGCTGC	55
		R: GCGGCATCGTTTGTGTGTAA	
TJ-7	(GT)_34_	F: HEX-GGCTCTGCTCGTGTGTTTTG	63.1
		R: GGGACTACACGGACTTCACC	
TJ-14	(GT)_27_	F: FAM-CTGGAGATCTGTGTCAGTGCA	54.5
		R: TGATGCAGCACACTCCAACA	
TJ-17	(CA)_27_	F: FAM-ATCCCCTTCTCCCTCACCTC	57
		R: GGTGTCTGTCCTTGTGCAGA	
TJ-20	(TG)_28_	F: HEX-GGGGTGGTGAGTGTGTGAAT	60
		R: GGACACACCTGCAGACAAGA	
TJ-21	(CCT)_20_	F: TET-GCCAGGGCCATTCTCTCTTT	57
		R: TTAGCTGCAGCACTTTGGGA	
TJ-18	(AC)_27_	F: FAM-TGTGTGTCTACTGAACGCCC	61
		R: TTTGCTGCCGTTCGACTTTC	

5’end of forward primer was labeled with different fluorescent dyes (FAM, HEX, NED, TET). *T*a is the optimal annealing temperature.

### Microsatellite loci genotyping and neutrality test

All *T*. *japonicus* samples were genotyped for the ten microsatellite loci: TJ-2, TJ-7, TJ-8, TJ-9, TJ-10, TJ-14, TJ-17, TJ-18, TJ-20, TJ-21 (Table 2). Microsatellite loci were amplified by PCR with primers designed above. Two PCR amplification conditions were applied: (1) For TJ-7, TJ-8, TJ-9, TJ-10, TJ-14, TJ-18, and TJ-21, the solution contained 10 μl Taq 2x Master Mix red (4 mM MgCl_2_) (Ampliqon, Denmark), 1 μl of template DNA, 0.5 μl of each primer, and ddH2O with a final volume of 20 μl; (2) For TJ-2, TJ-17, and TJ-20, the solution contained 10 μl SuperRed PCR Master Mix (2x) (4 mM MgCl_2_) (BIOTOOLS Co., Ltd, Taiwan), 1 μl of template DNA, 0.8 μl of each primer, and ddH2O with a final volume of 20 μl. Reactions had an initial step at 95°C for 3 min, followed by 40 cycles of denaturation at 94°C for 30 s, annealing at 54.5–66.5°C depending on the primers (Table 2) for 30 s and extension at 75°C for 45 s, and a final extension at 75°C for 5 min. Microsatellite DNA genotyping was performed using the 3730XL DNA Analyzer (Genomics, Taiwan). Genotypes were confirmed at Peak scanner v2.0 (ThermoFisher Scientific, USA), and the output microsatellite datasets were manually evaluated following Daniels et al. (1998) [62]. These datasets were then tested for genotyping errors, such as stuttering, allelic dropout, and presence of null alleles using MICRO-CHECKER v2.2.3 [63].

The departure of each microsatellite locus from Hardy-Weinberg equilibrium was assessed following procedures described by Louis and Dempster (1987) [64] and performed in GENEPOP [65, 66], with the *p-value* estimated by Markov Chain (MC) algorithm [67]. The default settings of the parameters were: dememorization number = 1000, batches = 100, and iterations = 1000. Detection of loci under selection based on F-statistics was performed in Arlequin 3.5 [68]. Coalescent simulations were used to obtain the *p-values* of locus-specific F-statistics conditioned on observed levels of heterozygosities [69, 70]. Unusual genetic differentiation levels will show if loci are detected under natural selection [69].

### Genetic diversity

Expected heterozygosity (H_E_) and observed heterozygosity (H_O_) were calculated for each sampling location following Weir and Cockerham (1984) [71] using GENETIX v4.05 [72]. Observed heterozygosity (H_O_) was directly calculated from the proportion of heterozygotes in the sample, whereas expected heterozygosity (H_E_) was calculated following Nei (1978) [73]. GENETIX v4.05 was also used to calculate fixation indices (F_ST_) and inbreeding coefficients (F_IS_) between and within each location based on the F-statistics method [71] with 1000 bootstrap replicates.

### Population structure

The potential genetic differentiation of *T*. *japonicus* was assessed through four methods. First, an unrooted neighbor-joining (NJ) phylogenetic tree was constructed for the five locations using the D_A_ distance method [74] with 1000 bootstrap replicates by POPTREE2 [75]. Second, Bayesian clustering was performed in STRUCTURE v.2.3.4 [76]. A burn-in period of 100,000× followed by 1,000,000× iterations on the Markov Chain Monte Carlo (MCMC) simulations was carried out. The number of separate clusters (K) was set from 1 to 5 and 10× iterations for each specific K-value. Then, the most probable K value based on the posterior probability was determined and subsequently used to assign each *T*. *japonicus* individual to its most probable cluster by Structure Harvester [77]. An *ad hoc* statistic of ΔK values was also calculated based on the rate of change of log probability of the data [78]. Third, Principal Coordinate Analysis (PCoA) was conducted through GenAlEx v.6.503 [79]. The inter-individual genetic distance of microsatellites loci was calculated following Smouse and Peakall (1999) [80] on the methods for codominant data. Fourth, the global and hierarchical partitioning of genetic structures were tested using the Analysis of Molecular Variance (AMOVA), setting codominant genotypic data for distance calculations, by GenAlEx with 9,999 permutations. Based on oceanographic features in the NW Pacific and the results of STRUCTURE v.2.3.4, we classified four types of hypothetical groupings for the five locations in the AMOVA analyses: (1) Five regions (DL; QD; ZH; GE; T); (2) Two regions (Yellow Sea: DL/QD; East China Sea and Taiwan Strait: ZH/GE/T); (3) Three regions (Yellow Sea: DL/QD; East China Sea: ZH/GE; Taiwan Strait: T); (4) Two regions (STRUCTURE results: QD; DL/ZH/GE/T).

### Effective population size, gene flow, and bottleneck effect

The effective population size (N_e_) and the number of migrants per generation were calculated based on the Maximum likelihood (ML) method in MIGRATE v1.2.32 [81]. N_e_ was inferred from the frequency of allele exchange among the locations and was calculated by the estimated population size parameter (mutation rate = 10^−3^) [82]. The assumption of a recently declined population, also known as a ‘bottleneck effect’, states that allele diversity reduces faster than heterozygosity due to the rapid loss of rare alleles [83, 84]. Compared to the expected heterozygosity in a population of constant size with the same number of alleles, a bottleneck effect signifies a temporary excess of heterozygosity [85, 86]. We used two tests to determine which population exhibits a significant number of loci with excess heterozygosity, namely the sign test [85] and Wilcoxon signed-rank test [87], which were both implemented in Bottleneck 1.2.02 [86]. The Wilcoxon signed-rank test is recognized as more powerful than the sign test and can produce better results for fewer polymorphic loci (n = 10 to 15) and datasets with a lower number of individuals (n = 15 to 40) [85]. The simulation result on the population containing a similar number of alleles was compared to the observed heterozygosity of a population evolving under the Infinite Allele Model (IAM) [88] and Two-Phase Model (TPM) [89]. The proportion of the Stepwise Mutation Model (SMM) in TPM was set to 5% and run for 100,000 iterations. The TPM model was hypothesized to fit with the microsatellite DNA data than that of the IAM model due to parameter explicitness [86, 90].

### Application of microsatellite markers in congeneric species

The application of the ten microsatellite markers developed in this study was also tested on three congeneric species, *T*. *brevis*, *T*. *lepturus*, and *T*. *nanhaiensis*, which also occur in the NW Pacific waters. Two DNA samples of each species were collected, extracted, and subjected to the PCR with conditions elaborated earlier.

## Results

### *De novo* RNA assembly and microsatellite loci identification

A total of 47,923,043 clean reads were generated after adaptor removal from the Illumina sequencing products. The length of assembled sequences ranged from 301 bp to 66,280 bp, with an average of 1,776 bp. GC content was 51.11%, and the length of N50 was 2,852 bp. Ensuing transcriptome assembly, a total of 46,632 assembled unigenes were identified. The microsatellite analysis using MISA examined the unigenes of *T*. *japonicus* transcriptome and found 40,027 (85.84%) that contained microsatellites. On all the motif repeat sizes, mononucleotide was the most abundant (41.29%) (S1 Table). A total of 10 pairs of primers designed in this study successfully amplified the targeted microsatellite regions, including 9 pairs for dinucleotide repeats and one pair for trinucleotide repeats (Table 2).

### Microsatellite genotyping and neutrality test

Genotyping errors caused by the presence of stutter and allele dropouts were not detected by MICRO-CHECKER. Null alleles were potentially present at the locus of TJ-2 in ZH and GE; TJ-9 in GE and T; TJ-10 in QD and GE; TJ-14 in ZH; TJ-18 in ZH and GE; and TJ-21 in GE and T, as suggested by the general excess of homozygotes for most allele size classes. Almost all loci showed a significant departure from Hardy-Weinberg equilibrium (*P < 0*.*05*) (S2 Table). Detection screening of loci under selection from F-statistics showed that none of the ten loci had unusual genetic differentiation levels, thus no sign of selection (S1 Fig). As the result, genotyping data of all ten loci were included for the following analyses (S3 Table).

### Genetic diversity

The number of alleles (N_a_), expected heterozygosity (H_E_), and observed heterozygosity (H_O_), per locus and location, were listed in S4 Table. The number of alleles ranged from 10 (TJ-17, ZH) to 36 (TJ-2, ZH and T), with an average of 23.14. Of the ten loci, TJ-2 (33.6) and TJ-17 (13.6) had the highest and lowest number of alleles, respectively. Of the five locations, GE (23.5) and ZH (22.9) had the highest and lowest number of alleles, respectively. The expected heterozygosity ranged from 0.8461 (TJ-17, ZH) to 0.9625 (TJ-2, ZH). The mean expected heterozygosity per locus was the highest in TJ-2 (0.95656) and lowest in TJ-17 (0.86978). The mean expected heterozygosity per location was highest at T (0.9253) while lowest at ZH (0.9156). The observed heterozygosity ranged from 0.7241 (TJ-18, ZH) to 0.9667 (TJ-7, QD). The mean observed heterozygosity per locus was the highest in TJ-7 (0.92), and the lowest in TJ-20 (0.84666). The mean observed heterozygosity per location was highest at QD (0.844) and lowest at GE (0.7939).

The F-statistics results of F_ST_ and F_IS_ were shown in S5 Table. The F_ST_ values ranged from -0.0043 (TJ-17, QD) to 0.01476 (TJ-18, T). For the F_ST_ values of the ten loci across all locations, the highest value was recorded from ZH (0.008019), while the lowest value was from DL (0.002937). All F_ST_ values were below 0.05, which indicated that there is no differentiation in each location. The F_IS_ values ranged from 0.00819 (TJ-14, QD) to 0.13604 (TJ-9, GE). For the F_IS_ values of ten loci in all five locations, the highest value was observed at DL (0.072342) and lowest at ZH (0.065282). All the F_IS_ values and the 95% confidence interval (0.04785–0.08527) were very low, indicating a low level of inbreeding in each location.

### Population structure

**Fig 2:**
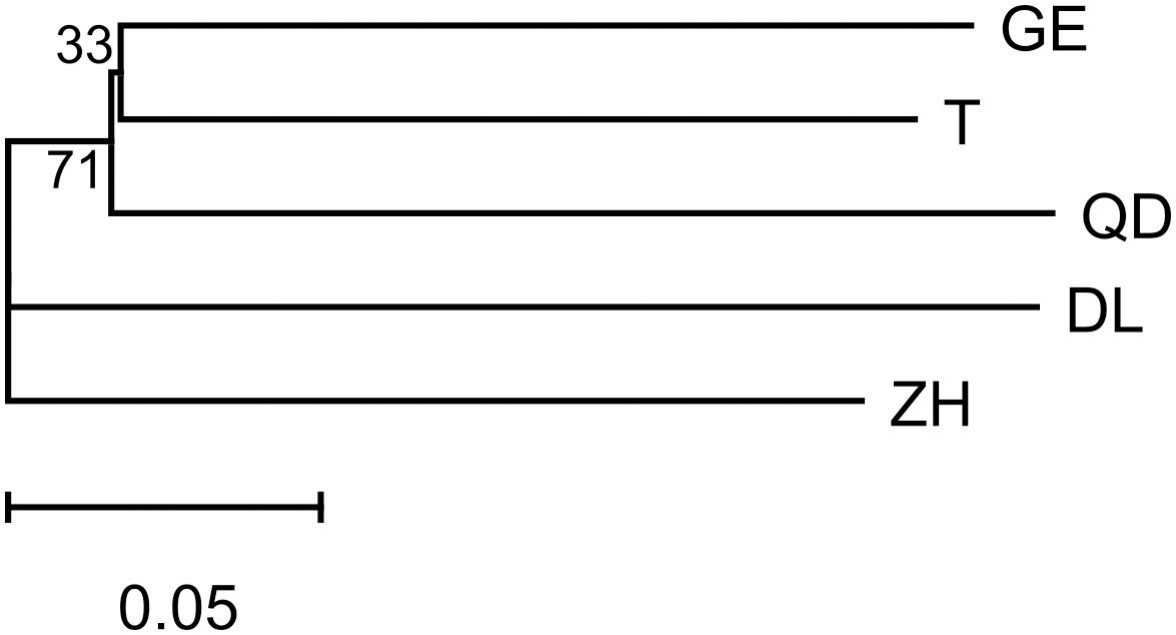
NJ tree based on ten microsatellite dataset from five *T*. *japonicus* populations. The scale bar at the bottom indicates the number of expected substitutions per site. Numbers by branch are bootstrap values.

**Fig 3:**
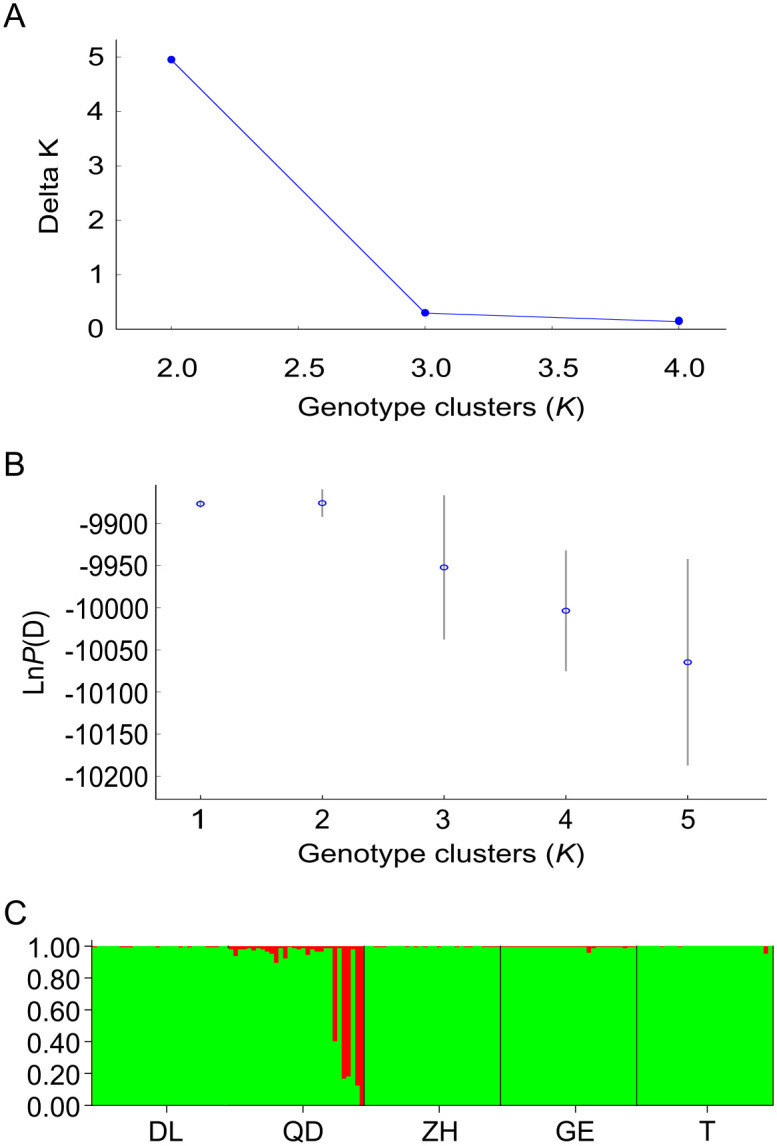
Hierarchical STRUCTURE analysis based on ten microsatellite datasets from 150 *T*. *japonicus* collected from five locations along the coasts of the NW Pacific. ΔK (A) and ln P(D) (B) are plotted against the number of genetic clusters (K). The population structure result when K = 2 is shown in (C). Each vertical colored line represents the assignment proportion of membership of each sample and categorized by locations.

**Fig 4:**
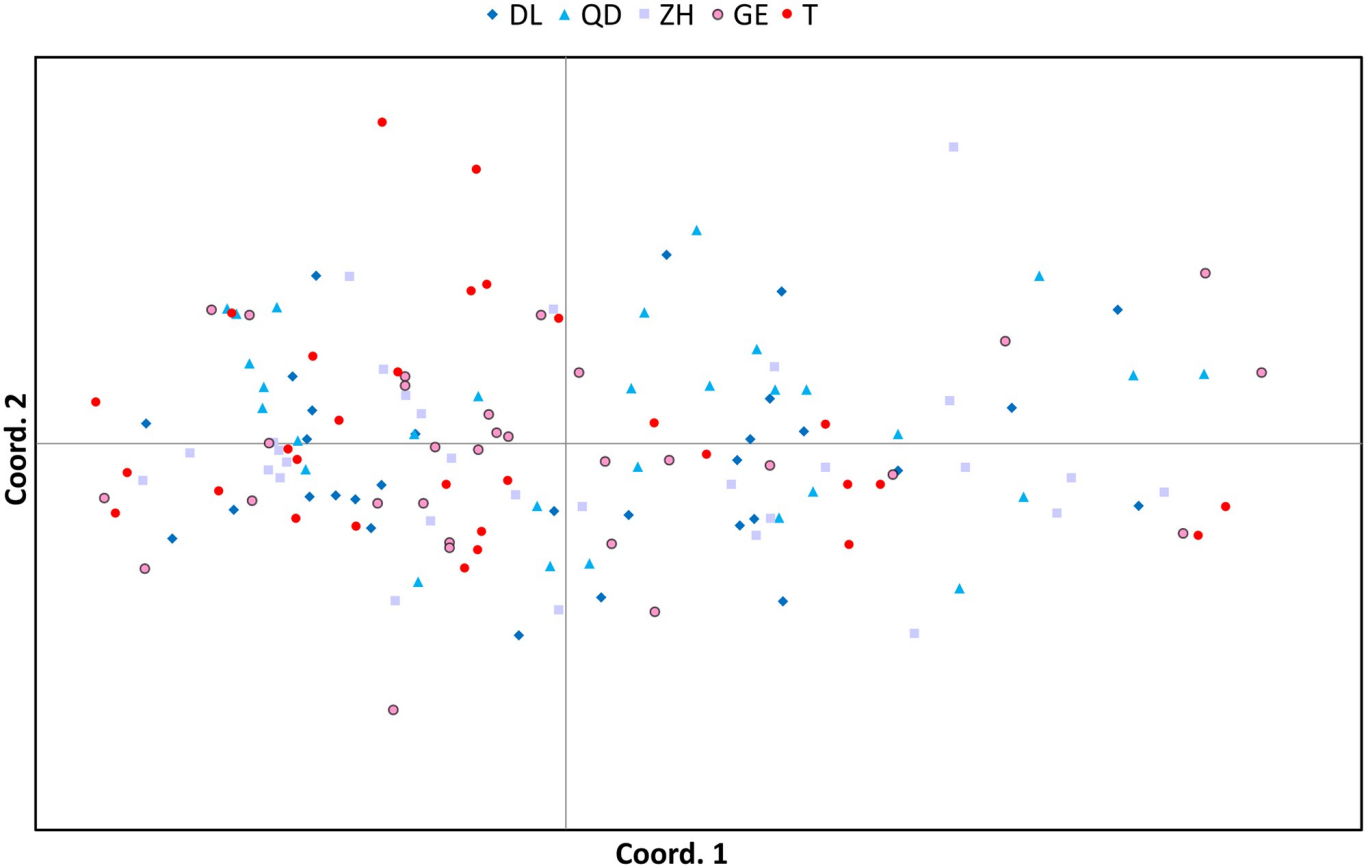
Plot of the first two axes of Principal Coordinate Analysis (PCoA) based on codominant genotypic distances of ten microsatellite loci for 150 *T*. *japonicus* individuals. Each dot represents an individual and is color-coded for the five locations reflecting latitudinal gradient (north: blue, south: red).

All four methods suggested a mixed population for *T*. *japonicus*. The NJ tree showed poor resolution on the genetic relationships of the five populations of *T*. *japonicus*, represented with low node supporting values (Fig 2). STRUCTURE Harvester showed that ΔK was highest at K = 2 (Fig 3A), with the most probable K value at K = 1 and 2 (Fig 3B). The result of K = 1 suggested that the five locations were not further separated into different clusters. Meanwhile, the result of K = 2 showed that the majority of individuals from all locations comprise a large single cluster, while few individuals from QD are categorized into a smaller second cluster (Fig 3C). The PCoA result based on co-dominant genotypic distances revealed that all individuals from five locations are mixed (Fig 4). The first three axes accounted for 7.99% of the total variation, and 2.94%, 2.56%, 2.49% for each. The pairwise F_ST_ values among locations ranged from 0.00148 (between GE and T) to 0.00766 (between DL and QD). They were significantly different for DL/QD/ZH vs T; QD vs DL; ZH vs T; and ZH vs the rest of the locations (S6 Table). AMOVA results indicated that there was no differentiation among locations under the four types of hypothetical groupings. The percentages of total variation among regions were all less than 1%, and most of the genetic variations were within locations with percentages higher than 98% (S7 Table).

### Effective population size, gene flow, and bottleneck effect

The effective population size (N_e_) of DL, QD, ZH, GE, and T were 21,343.79; 24,014.94; 19,529.13; 20,971.69; and 22,655.89, respectively. The number of migrants per generation ranged from 15,739.67 (DL to T) to 19,656.04 (GE to QD) (S8 Table)—more than half of the effective population size per generation—which showed significant migration activities between locations. Upon ranking these migrant estimates, QD appeared to be the highly-migrated sampling location by all the other populations. Also, all ten loci fit both the IAM and TPM mutation models. According to the sign test, all locations except QD had significant heterozygosity excess under the IAM model; only T had significant heterozygosity excess under the TPM model. According to the Wilcoxon signed-rank test, all locations had significant heterozygosity excess under the IAM model; all locations except QD had significant heterozygosity excess under the TPM model (Table 3).

**Table 3:** Bottleneck effect tests for five populations of *T*. *japonicus*.

	IAM		TPM	
	Sign test	Wilcoxon Test	Sign test	Wilcoxon Test
DL	0.00643*	0.00049***	0.19402	0.00928*
QD	0.18151	0.00684*	0.35077	0.3125
ZH	0.04942*	0.00146**	0.10466	0.01611*
GE	0.00633*	0.00049***	0.05418	0.00928*
T	0.00727*	0.00049***	0.00715*	0.00049*

IAM: Infinite Allele Model; TPM: Two-Phase Mutation model.

*P<0.05,

**P<0.005,

***P<0.001.

### Application of microsatellite markers in congeneric species

The ten microsatellite markers developed from *T*. *japonicus* were highly successful in cross-amplifying with the three congeneric species (S2 Fig). For all three species, TJ-2, -7, -10, -14, -20, and -21 were most promising by showing two clear bands around the desired PCR size range in the gel photo. Additionally, TJ-8, -9, and -18 were promising exclusively for *T*. *lepturus* and *T*. *nanhaiensis*. However, some adjustments in PCR conditions (i.e., screening for optimum annealing temperature) were required for amplifying the pure target products in some cases.

## Discussion

### EST-microsatellite markers

Genome-wide genetic information can help us better understand the biological characteristics and molecular mechanisms of organisms. In recent years, Next Generation Sequencing (NGS) has become an efficient and cost-effective method to generate genome-scale sequence data, which advanced the formulation of molecular markers for both model and non-model organisms [91]. Cutlassfish is a non-model organism, and its genome data is currently unavailable. In this study, we generated the first cutlassfish transcriptome dataset from the highly exploited *T*. *japonicus* in the NW Pacific using one of the NGS platforms—Illumina, which will lay the foundation for future related studies.

Molecular markers are an important tool to study population genetic diversity. Microsatellite DNA markers are particularly useful in monitoring genetic variability due to their inherent high mutation rates, thus have also been successfully applied to the management of fisheries species [92]. In this study, we identified 40,027 microsatellite markers from the transcriptome dataset. Similar to other fish species, the high proportion of identified microsatellite DNA markers (41.29%) were mononucleotide repeats [40, 93–95].

The null hypothesis on genetic studies states that populations are selectively neutral, meaning they are not influenced by natural selection but by random fixation [96, 97]. Therefore, the departure from Hardy-Weinberg equilibrium of the loci due to selection might not be an ideal marker for population genetic studies. Out of the final ten microsatellite loci applied in this study, some significantly departed from Hardy-Weinberg equilibrium due to heterozygote deficiencies. Probable reasons could include genetic drift, inbreeding, selection, and the presence of null alleles. However, these probabilities were ruled out due to a lack of justification. Since genetic drift often has more impacts on small populations [98], this becomes unlikely for *T*. *japonicus* being the dominant cutlassfish species in the NW Pacific and the estimated effective population size per sampling locality also ranged between 19,529 to 24,014. Inbreeding is neither a feasible reason because the inbreeding coefficients are positive yet very low [99]. We did not see evidence of either natural or human-induced selection, such as fishing pressure, based on the F-statistics results. A microsatellite null allele can be derived from a failed amplification during the PCR process [100]. In this study, some potential null alleles were suggested yet their homozygous identity was validated by reperforming the PCR using lowered annealing temperature. Carlsson (2008) also showed that despite the presence of null alleles, this does not necessarily contribute a big impact on the subsequent analyses, compared to other factors such as the strength of population differentiation and the number of loci [100].

Another potential explanation for the departures from Hardy-Weinberg equilibrium is the Wahlund effect [101]. In population genetics, Wahlund effect is the reduction of heterozygosity in a population due to the presence of subpopulation separations by geographic barriers or age structure [102]. Cutlassfish in the NW Pacific have multiple spawning seasons and multiple spawning sites [9, 103] contributing to temporal and spatial subpopulations [102, 104]. If two or more subpopulations have unique allele frequencies, the overall heterozygosity would be lower than expected—assuming there is a random mating population [101]. Wahlund effect should be expected for the mixed-age population at individual loci [102], such as in *T*. *japonicus*.

Finally, we tried to remove loci with departures from the Hardy-Weinberg equilibrium from multiple locations, repeated all analyses, and still obtained the same results. Therefore, we consider that these non-neutral loci might have a weak influence on our subsequent analyses. Also, the inclusion of multiple loci would help average the effects of selection [105]; therefore, all the ten microsatellite markers were retained for the succeeding population genetic analyses.

### High genetic diversity

Genetic diversity indices, including the number of alleles and observed heterozygosities of the ten microsatellite markers showed higher values in *T*. *japonicus* than other reports on commercial marine fishes. The average number of alleles per locus of *T*. *japonicus* (23.14) was relatively higher than that of striped bass *Morone saxatilis* (14.125) [42], pompano *Trachinotus ovatus* (5.875) [93], and small yellow croaker *Larimichthys polyactis* (5.82) [106]. Whether these differences might be species or marker-related, the average number of alleles per locus of *T*. *japonicus* was relatively higher than 12 other marine fish species (20.6) based on 66 loci [107]. The average observed heterozygosity per locus (0.82514) also showed the same findings, which is higher than other commercial fishes, such as striped bass *Morone saxatilis* (0.766) [42], pompano *Trachinotus ovatus* (0.318) [93], and yellow drum *Nibea albiflora* (0.8033) [108]; and also higher than the average of marine fishes (0.79) [107]. Among the published microsatellite genetic parameters of cutlassfish species, the average number of alleles per locus (23.14) and the average observed heterozygosity per locus (0.82514) of *T*. *japonicus* were higher than other cutlassfish species such as *T*. *haumela* (N_a_ = 4.58, H_o_ = 0.661; N_a_ = 12.3, H_o_ = 0.71) [45, 109], *T*. *lepturus* (N_a_ = 11.6, H_o_ = 0.804) [46], *T*. *nanhaiensis* (N_a_ = 4.4, H_o_ = 0.807) [47], and *L*. *savala* (N_a_ = 9.11, H_o_ = 0.634; N_a_ = 12.5, H_o_ = 0.81) [49, 50].

The high genetic diversity indices typically indicate a larger population size and/or higher mutation rate [110]. Since mutation rates should be similar among closely related species [111], a larger population size could be responsible for the relatively higher genetic diversity of *T*. *japonicus*. In the NW Pacific, *T*. *japonicus* has the widest distribution range compared to its congeners [6] and has been reported as the dominant cutlassfish species [7, 112], with an estimated effective population size per location of around ~20,000. In addition, Hsu et al. (2007) proposed that the high genotypic differentiation among cohorts is the underlying mechanism for the genetic diversity in cutlassfish [113]. This differentiation was contributed by the high fecundities and larval mortalities of the species, which in response increase the variance in reproductive success for different cohorts [114]. This heterozygosity is commonly used as an indicator of the informational power of a marker. The heterozygosity values per locus ranged from 0.84666 to 0.92 for the ten microsatellite markers designed in this study, thus indicating high polymorphicity and ideal for population genetic analyses [115].

### A well-mixed population experienced bottleneck effect

The results of the NJ tree, AMOVA, PCoA, and STRUCTURE all suggested a single well-mixed population of *T*. *japonicus* in the NW Pacific. The NJ tree yielded poor phylogenetic resolution to separate populations, which was indicative of a homogenous structuring. AMOVA showed that there was no significant differentiation at all geographic hierarchical levels. PCoA displayed that all samples were mixed in the quadrant and no obvious grouping was evident. The results of STRUCTURE showed that both K = 1 and K = 2 population schemes were the most probable; however, only a few individuals from Qingdao were assigned to the second cluster, and the reason behind that is still unknown. Although differentiation among some locations was statistically significant based on F-statistics, the F_ST_ values were all much lower than 0.05 suggesting minimal differentiation [99]. In addition, the number of migrants per generation among locations was more than half of the effective population size which can greatly facilitate the admixture. The slightly higher migration preference towards Qingdao might explain its relatively high observed heterozygosity. Since populations prefer migrating to Qingdao, this location might have become a genetic pseudosink. Pseudosinks are characterized as viable habitats, particularly due to the very high immigration rate [116, 117]. We suspect that this migratory preference might be related to habitat productivity. Qingdao particularly lies adjacent to the Bohai Sea-Yellow Sea boundary. Nutrient inputs carried by the intrusive YSWC might be partially stalled at the narrow Bohai Sea opening and promotes habitat productivity. In addition, the nutrients discharged in the Bohai Sea and Huanghe River, flushed through by the Bohai Sea Coastal Current also girds around the Shandong coast [118, 119]–where Qingdao is particularly located. This dynamic potentially makes Qingdao an ideally productive foraging ground as reflected also by numerous eutrophication reports [119–121]. This overall high dispersal behavior of *T*. *japonicus* was also previously reported to be seasonal in the China Seas and potentially related to spawning seasons [5]. Our finding of a single well-mixed population of *T*. *japonicus* is congruent with previous genetic results using mitochondrial DNA markers [25, 26]. This is likely attributed to the absence of geographic isolation in the NW Pacific and the great migratory ability of this fish. Marine fishes can have low levels of genetic differentiation among locations because of the relatively high dispersal potential during pelagic egg, larval, or even adult stages and lack of physical barriers to the movement [122, 123].

A recent bottleneck event was proposed for the majority, if not all locations, based on the sign tests. Although the timing of this event was not estimated, we suspected that it corresponded to the sea-level change in the Pleistocene. It had been well recognized that the cyclical fluctuations of the global climate in the Pleistocene caused drastic changes in sea level, which can affect the distribution range and biomass of marine organisms [123, 124]. This sea-level change can shape the population history of marine species [25, 125, 126]. In addition, biological characteristics of species such as dispersal ability and oceanic characteristics of the environment also contribute to the genetic structure of marine organisms [127, 128]. During the glacial period, the coastline was pushed offshore, and marine residents were forced to new geographic areas or refugia. For continental shelf species in the NW Pacific such as cutlassfishes, suitable habitat was reduced to an elongated enclosed sea in the East China Sea and the South China Sea (the Okinawa Trough) with an area <1/3 of its present size [129], which served as a refuge for the survivors [130].

The Bohai Bay, the Yellow Sea, and the Taiwan Strait all disappeared completely. After the glacial period, rising sea levels brought the fauna back to a broader habitat and open opportunities to expand the population size. He, et al. (2014) and Xiao, et al. (2014) proposed a population expansion of *T*. *japonicus* in the late Pleistocene based on mitochondrial DNA markers [25, 26]. The great dispersal ability of cutlassfish and the absence of prominent oceanographic barriers in this area greatly facilitate the expansion process. This also explains the lack of significant population genetic differentiation in the continental shelf of China, which has been reported in taxon-wide marine organisms, including shrimps, polychaete worms, gastropods, and fish [130].

### Fishery management

A downward trend of the total catch of cutlassfish in the NW Pacific area has been reported [131–134]. Some studies suggested that persisting fishing pressure had changed their growth and reproduction pattern, as seen in their precocious maturity and miniaturization [5, 8, 135], and this led to fitness reduction [136]. In this study, we found that *T*. *japonicus* has high genetic diversity and large population size, suggesting that this highly exploited species is resilient to previous impacts. Moreover, a single population with high gene flow across locations had been identified throughout the NW Pacific. Recent fisheries data have shown that the top exploited Trichiurid relative, *T*. *lepturus*, has already been experiencing a decreasing catch rate since the last decade [132]. This serves as a warning and calls for urgent *T*. *japonicus* management schemes as this species could potentially be the next sought target.

Based on the above genetic characteristics, we suggest recognizing the entire sampling localities as a single fishery unit and developing international cooperative management. More stringent measures can also be applied to fishing grounds with relatively higher recorded heterozygosity and migration preference (i.e., Qingdao) to establish more substantial protection for the existing genetic diversity. The successful amplification of the EST-microsatellite markers developed in this study towards congeneric species implies their usefulness for characterizing genetic diversity on other highly targeted Trichiurids and confirms the advantage of transferability across related species which economized time and resources [38]. With these cross-amplified markers, it can pave the way for the establishment of genetic marker repositories for the vulnerable Trichiurid species from the NW Pacific.

## Conclusions

This is the first study applying microsatellite markers to depict the population genetics of *T*. *japonicus*. The ten microsatellite loci developed from transcriptome displayed high polymorphism and genetic diversity thus proved as ideal markers to study intra-specific variations. We found that *T*. *japonicus* has higher genetic diversity than other marine fishes including the cutlassfish relatives, which might be attributed to its large population size and/or cohort genetic differentiation. Besides, a well-mixed single population was identified for *T*. *japonicus* in the NW Pacific, which has experienced a bottleneck and subsequent expansion likely facilitated by the strong migratory ability and the lack of dispersal barriers. High heterozygosity and migration preference were also reflected for Qingdao, and hence considered as a genetic pseudosink. Based on the above genetic characteristics, we suggest treating the entire NW Pacific population as one fishery unit and developing international cooperative management for the species. The developed microsatellite markers have proven effectiveness for transferability in congeneric species investigations.

## Supporting information

S1 Fig F_ST_ (small circles) for individual microsatellite locus plotted against heterozygosity. Dashed lines are one-sided confidence interval limits obtained from simulation-based expected distributions of F_ST_ assuming a stepwise mutation mode.
(DOCX)

S2 Fig Gel photo of the PCR results of the ten microsatellite markers in four *Trichiurus* species (Tj: *T*. *japonicus*, Tb: *T*. *brevis*, Tl: *T*. *lepturus*, and Tn: *T*. *nanhaiensis*). (DOCX)

S1 Table The total number and percentage of simple sequence repeats (SSRs) identified for each motif length. (DOCX)

S2 Table The results of Hardy-Weinberg equilibrium analyses for each locus in five locations. (DOCX)

S3 Table The genotyping results of each individual. (XLSX)

S4 Table Genetic diversity estimated based on the ten microsatellite loci in five populations of *T*. *japonicus*. Na: number of alleles; He: expected heterozygosity; Ho: observed heterozygosity. Dark gray means number of alleles > 30 and heterozygosity > 0.9; medium gray means number of alleles between 20 to 30 and heterozygosity between 0.85 to 0.9; light grey means number of alleles < 20 and heterozygosity < 0.85.
(DOCX)

S5 Table The F-statistics index estimated based on the ten microsatellite loci in five populations of *T*. *japonicus*. F_ST_: The fixation index; F_IS_: The Wright’s fixation index.
(DOCX)

S6 Table Matrix of pairwise F_ST_ among five populations based on *T*. *japonicus* microsatellite data. (DOCX)

S7 Table Analysis of molecular variance (AMOVA) results for the four types of groupings. d.f., degree of freedom.
(DOCX)

S8 Table The number of migrants per generation among locations. N_m_ = Number of migrants. Location 1 = DL, 2 = QD, 3 = ZH, 4 = GE, and 5 = T.
(DOCX)

S1 Raw images (PDF)
