# Supplementary material for: High migratory propensity constitutes a single stock of an exploited cutlassfish species in the Northwest Pacific: A microsatellite approach
Source: PLoS One. 2022 Mar 17;17(3):e0265548. doi: 10.1371/journal.pone.0265548 (PMC8929604; doi:10.1371/journal.pone.0265548)
Supplement: S1 Fig — Dashed lines are one-sided confidence interval limits obtained from simulation-based expected distributions of FST assuming a stepwise mutation mode. (DOCX) [file pone.0265548.s001.docx]

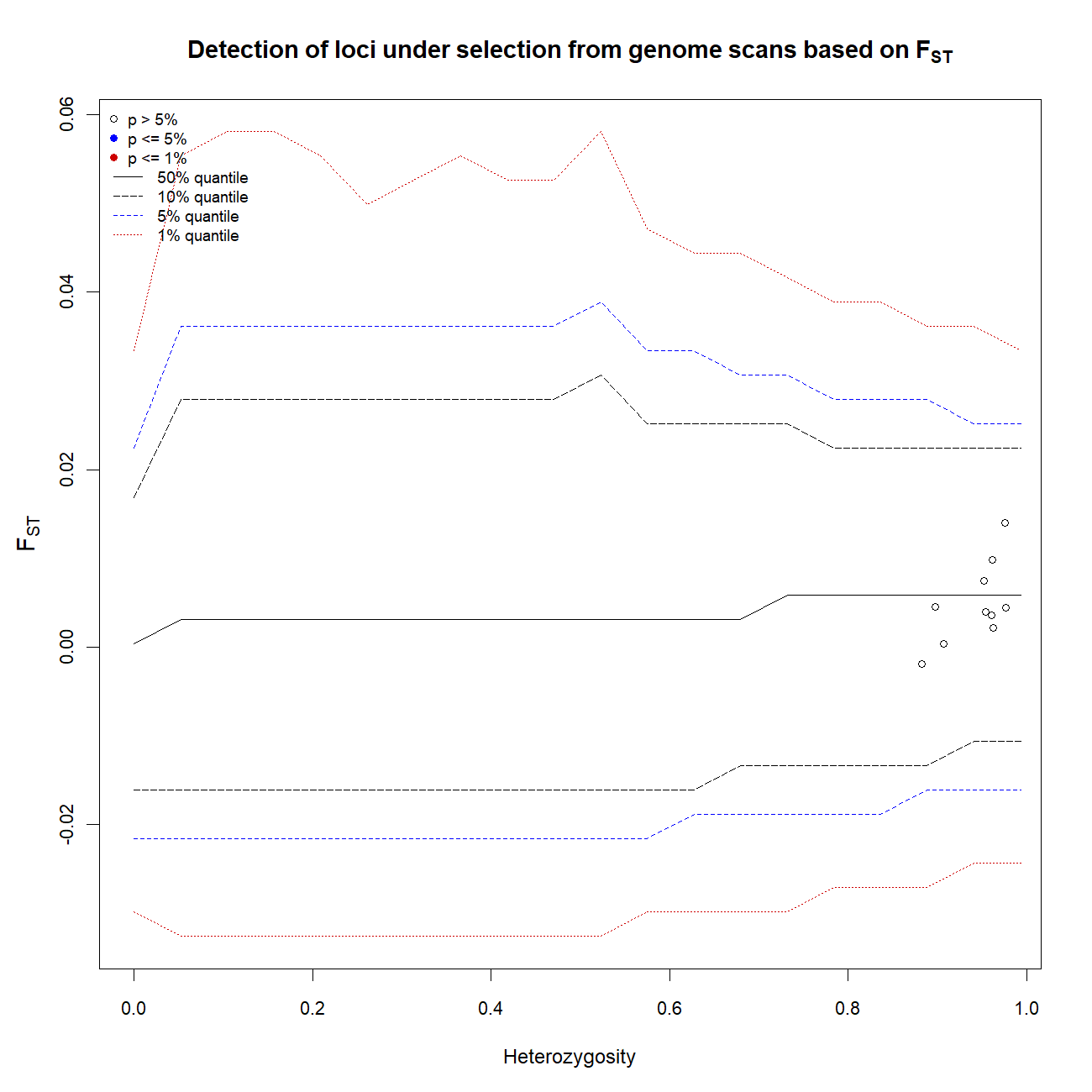


S1 Fig. F_ST_ (small circles) for individual microsatellite locus plotted against heterozygosity. Dashed lines are one-sided confidence interval limits obtained from simulation-based expected distributions of F_ST_ assuming a stepwise mutation mode.
