# Supplementary material for: High migratory propensity constitutes a single stock of an exploited cutlassfish species in the Northwest Pacific: A microsatellite approach
Source: PLoS One. 2022 Mar 17;17(3):e0265548. doi: 10.1371/journal.pone.0265548 (PMC8929604; doi:10.1371/journal.pone.0265548)

S2 Fig. Gel photo of the PCR results of the ten microsatellite markers in four *Trichiurus* species (Tj: *T. japonicus*, Tb: *T. brevis*, Tl: *T. lepturus*, and Tn: *T. nanhaiensis*).


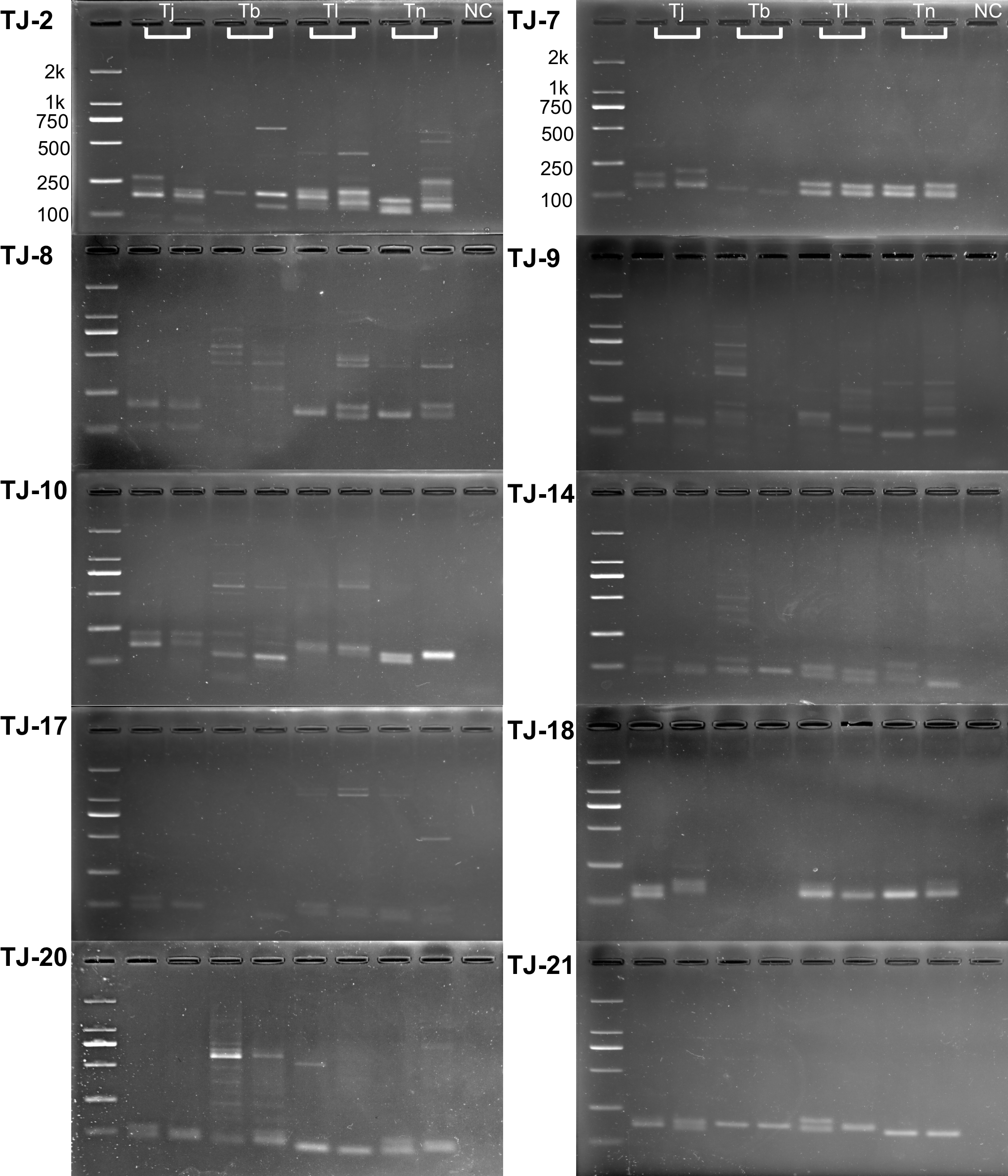

Supplement: S2 Fig — (DOCX) [file pone.0265548.s002.docx]
