# Supplementary material for: High migratory propensity constitutes a single stock of an exploited cutlassfish species in the Northwest Pacific: A microsatellite approach
Source: PLoS One. 2022 Mar 17;17(3):e0265548. doi: 10.1371/journal.pone.0265548 (PMC8929604; doi:10.1371/journal.pone.0265548)
Supplement: S1 Table — (DOCX) [file pone.0265548.s003.docx]

S1 Table. The total number and percentage of simple sequence repeats (SSRs) identified for each motif length.

|  | Number of SSRs | Percentage (%) |
| --- | --- | --- |
| Mono- | 16529 | 41.29 |
| Di- | 13355 | 33.36 |
| Tri- | 9510 | 23.76 |
| Tetra- | 571 | 1.43 |
| Penta- | 38 | 0.09 |
| Hexa- | 24 | 0.07 |
