# Supplementary material for: High migratory propensity constitutes a single stock of an exploited cutlassfish species in the Northwest Pacific: A microsatellite approach
Source: PLoS One. 2022 Mar 17;17(3):e0265548. doi: 10.1371/journal.pone.0265548 (PMC8929604; doi:10.1371/journal.pone.0265548)
Supplement: S2 Table — (DOCX) [file pone.0265548.s004.docx]

S2 Table. The results of Hardy-Weinberg equilibrium analyses for each locus in five locations.

|  | CH_DL | CH_QD | CH_ZH | TW_GE | TW_T |
| --- | --- | --- | --- | --- | --- |
| TJ-2 | 0.2706 | 0.154 | 0* | 0.0039* | 0.2837 |
| TJ-7 | 0.6224 | 0.7155 | 0.5416 | 0.2726 | 0.3976 |
| TJ-8 | 0.0291* | 0* | 0.0249* | 0.2866 | 0.1429 |
| TJ-9 | 0.0103* | 0.2139 | 0.0576 | 0* | 0.0029* |
| TJ-10 | 0.4264 | 0.0183* | 0.4366 | 0.3362 | 0* |
| TJ-14 | 0.0497* | 0.4185 | 0.0088* | 0.7125 | 0.5932 |
| TJ-17 | 0.1132 | 0.4822 | 0.0192* | 0.1914 | 0.023* |
| TJ-18 | 0* | 0.3564 | 0.0019* | 0* | 0.0887 |
| TJ-20 | 0.0005* | 0.0713 | 0.1501 | 0.4307 | 0.1968 |
| TJ-21 | 0* | 0.0592 | 0.3462 | 0.0594 | 0.0104* |

* P < 0.05
