# Supplementary material for: High migratory propensity constitutes a single stock of an exploited cutlassfish species in the Northwest Pacific: A microsatellite approach
Source: PLoS One. 2022 Mar 17;17(3):e0265548. doi: 10.1371/journal.pone.0265548 (PMC8929604; doi:10.1371/journal.pone.0265548)
Supplement: S4 Table — Na: number of alleles; He: expected heterozygosity; Ho: observed heterozygosity. Dark gray means number of alleles > 30 and heterozygosity > 0.9; medium gray means number of alleles between 20 to 30 and heterozygosity between 0.85 to 0.9; light grey means number of alleles < 20 and heterozygosity < 0.85. (DOCX) [file pone.0265548.s006.docx]

S4 Table. Genetic diversity estimated based on the ten microsatellite loci in five populations of *T. japonicus.* Na: number of alleles; He: expected heterozygosity; Ho: observed heterozygosity. Dark gray means number of alleles > 30 and heterozygosity > 0.9; medium gray means number of alleles between 20 to 30 and heterozygosity between 0.85 to 0.9; light grey means number of alleles < 20 and heterozygosity < 0.85.

|  |  | TJ-2 | TJ-7 | TJ-8 | TJ-9 | TJ-10 | TJ-14 | TJ-17 | TJ-18 | TJ-20 | TJ-21 | Mean |
| --- | --- | --- | --- | --- | --- | --- | --- | --- | --- | --- | --- | --- |
| CH_DL | Na | 35 | 24 | 27 | 27 | 26 | 21 | 14 | 31 | 13 | 12 | 23 |
|  | He | 0.9572 | 0.9394 | 0.9411 | 0.9356 | 0.9461 | 0.92 | 0.8589 | 0.9572 | 0.8656 | 0.8672 | 0.9197 |
|  | Ho | 0.9 | 0.9333 | 0.9 | 0.8667 | 0.9333 | 0.8667 | 0.9 | 0.9 | 0.9 | 0.8667 | 0.8387 |
| CH_QD | Na | 29 | 26 | 24 | 26 | 28 | 23 | 16 | 24 | 19 | 15 | 23 |
|  | He | 0.9478 | 0.9422 | 0.92 | 0.9362 | 0.9433 | 0.9178 | 0.9033 | 0.9456 | 0.8756 | 0.8933 | 0.9178 |
|  | Ho | 0.9 | 0.9667 | 0.8667 | 0.8889 | 0.8667 | 0.9333 | 0.9 | 0.9 | 0.8 | 0.9 | 0.844 |
| CH_ZH | Na | 36 | 24 | 25 | 31 | 24 | 24 | 10 | 23 | 18 | 14 | 22.9 |
|  | He | 0.9625 | 0.9428 | 0.9333 | 0.9517 | 0.9429 | 0.9467 | 0.8461 | 0.915 | 0.8839 | 0.8789 | 0.9156 |
|  | Ho | 0.8276 | 0.9 | 0.9 | 0.9 | 0.931 | 0.8667 | 0.8333 | 0.7241 | 0.9 | 0.9333 | 0.8247 |
| TW_GE | Na | 32 | 24 | 22 | 28 | 24 | 27 | 14 | 31 | 16 | 17 | 23.5 |
|  | He | 0.9542 | 0.9306 | 0.9339 | 0.9477 | 0.9317 | 0.9344 | 0.8789 | 0.9533 | 0.8811 | 0.9094 | 0.9208 |
|  | Ho | 0.7931 | 0.9 | 0.9333 | 0.7857 | 0.9333 | 0.9333 | 0.8667 | 0.8333 | 0.8333 | 0.7667 | 0.7939 |
| TW_T | Na | 36 | 22 | 26 | 28 | 22 | 23 | 14 | 33 | 13 | 16 | 23.3 |
|  | He | 0.9611 | 0.9289 | 0.9453 | 0.9483 | 0.94 | 0.93 | 0.8617 | 0.9598 | 0.8872 | 0.91 | 0.9253 |
|  | Ho | 0.9333 | 0.9 | 0.8966 | 0.8571 | 0.8276 | 0.9333 | 0.8333 | 0.9286 | 0.8 | 0.7667 | 0.8244 |
| Mean | Na | 33.6 | 24 | 24.8 | 28 | 24.8 | 23.6 | 13.6 | 28.4 | 15.8 | 14.8 | 23.14 |
|  | He | 0.95656 | 0.93678 | 0.93472 | 0.9439 | 0.9408 | 0.92978 | 0.86978 | 0.94618 | 0.87868 | 0.89176 | 0.91984 |
|  | Ho | 0.8708 | 0.92 | 0.89932 | 0.85968 | 0.89838 | 0.90666 | 0.86666 | 0.8572 | 0.84666 | 0.84668 | 0.82514 |
