# Supplementary material for: High migratory propensity constitutes a single stock of an exploited cutlassfish species in the Northwest Pacific: A microsatellite approach
Source: PLoS One. 2022 Mar 17;17(3):e0265548. doi: 10.1371/journal.pone.0265548 (PMC8929604; doi:10.1371/journal.pone.0265548)
Supplement: S5 Table — FST: The fixation index; FIS: The Wright’s fixation index. (DOCX) [file pone.0265548.s007.docx]

S5 Table. The F-statistics index estimated based on the ten microsatellite loci in five populations of *T. japonicus.* F_ST_: The fixation index; F_IS_: The Wright’s fixation index.

|  |  | TJ-2 | TJ-7 | TJ-8 | TJ-9 | TJ-10 | TJ-14 | TJ-17 | TJ-18 | TJ-20 | | TJ-21 | | Average | |
| --- | --- | --- | --- | --- | --- | --- | --- | --- | --- | --- | --- | --- | --- | --- | --- |
| CH_DL | F_IS_ | 0.11322 | 0.03771 | 0.05336 | 0.11059 | 0.06998 | 0.03363 | 0.03318 | 0.12024 | 0.072 | 0.07951 | | 0.072342 | |  |
|  | F_ST_ | 0.00287 | 0.00844 | 0.0053 | -0.00215 | 0.0037 | -0.00029 | -0.0042 | 0.01392 | 0.00541 | | -0.00363 | | 0.002937 | |
| CH_QD | F_IS_ | 0.11548 | 0.04588 | 0.04988 | 0.11493 | 0.0527 | 0.00819 | 0.02066 | 0.12307 | 0.04094 | | 0.08199 | | 0.065372 | |
|  | F_ST_ | 0.00137 | 0.00999 | 0.00305 | 0.00031 | 0.00256 | 0.00565 | -0.0043 | 0.0127 | 0.00323 | | -0.00223 | | 0.003233 | |
| CH_ZH | F_IS_ | 0.09313 | 0.02793 | 0.05538 | 0.11584 | 0.06969 | 0.02655 | 0.0179 | 0.08429 | 0.06707 | | 0.09504 | | 0.065282 | |
|  | F_ST_ | 0.00315 | 0.00867 | 0.00023 | 0.00236 | 0.00064 | 0.05206 | -0.00091 | 0.00657 | 0.00771 | | -0.00029 | | 0.008019 | |
| TW_GE | F_IS_ | 0.08633 | 0.06421 | 0.10227 | 0.13604 | 0.07366 | 0.04773 | 0.01807 | 0.10315 | 0.04887 | | 0.04025 | | 0.072058 | |
|  | F_ST_ | 0.00382 | 0.00887 | 0.00161 | -0.00012 | 0.00031 | 0.00949 | 0.00024 | 0.01274 | 0.006 | | 0.00148 | | 0.004444 | |
| TW_T | F_IS_ | 0.12113 | 0.05134 | 0.07853 | 0.10451 | 0.04367 | 0.04889 | 0.01345 | 0.12574 | 0.03769 | | 0.0401 | | 0.066505 | |
|  | F_ST_ | 0.00166 | 0.005 | 0.00703 | 0.00039 | 0.00549 | 0.00598 | -0.00202 | 0.01476 | -0.00439 | | 0.00015 | | 0.003405 | |
