# Supplementary material for: High migratory propensity constitutes a single stock of an exploited cutlassfish species in the Northwest Pacific: A microsatellite approach
Source: PLoS One. 2022 Mar 17;17(3):e0265548. doi: 10.1371/journal.pone.0265548 (PMC8929604; doi:10.1371/journal.pone.0265548)
Supplement: S6 Table — (DOCX) [file pone.0265548.s008.docx]

S6 Table. Matrix of pairwise F_ST_ among five populations based on *T. japonicus* microsatellite data.

|  | DL | QD | ZH | GE | T |
| --- | --- | --- | --- | --- | --- |
| DL | ------------ |  |  |  |  |
| QD | 0.00766* | ------------- |  |  |  |
| ZH | 0.00453* | 0.00481* | ------------- |  |  |
| GE | 0.00447 | 0.00454 | 0.00511* | --------------- |  |
| T | 0.00591* | 0.00383* | 0.00477* | 0.00148 | -------------- |

*: P < 0.05
