# Supplementary material for: High migratory propensity constitutes a single stock of an exploited cutlassfish species in the Northwest Pacific: A microsatellite approach
Source: PLoS One. 2022 Mar 17;17(3):e0265548. doi: 10.1371/journal.pone.0265548 (PMC8929604; doi:10.1371/journal.pone.0265548)
Supplement: S7 Table — d.f., degree of freedom. (DOCX) [file pone.0265548.s009.docx]

S7 Table. Analysis of molecular variance (AMOVA) results for the four types of groupings. d.f., degree of freedom

| Source of variation | d.f. | Sum of squares (SS) | Mean squares (MS) | Estimate of variance | Percentage of total variation (%) | *P* |
| --- | --- | --- | --- | --- | --- | --- |
| Type 1: DL vs QD vs ZH vs GE vs T | | | | | | |
| Among populations | 4 | 48.661 | 12.165 | 0.071 | 0.707% | 0.001 |
| Within populations | 145 | 1453.483 | 10.024 | 10.024 | 99.293% |  |
| Total | 149 | 1502.144 |  | 10.095 | 100% |  |
|  |  |  |  |  |  |  |
| Type 2: DL/QD vs ZH/GE/T | | | | | | |
| Among regions | 1 | 11.475 | 11.475 | 0.000 | 0.000% | 0.760 |
| Among population | 3 | 37.186 | 12.395 | 0.079 | 0.782% | 0.002 |
| Within populations | 145 | 1453.483 | 10.024 | 10.024 | 99.218% | 0.003 |
|  |  |  |  |  |  |  |
| Type 3: DL/QD vs ZH/GE vs T | | | | | | |
| Among regions | 2 | 22.312 | 11.156 | 0.000 | 0.000% | 0.974 |
| Among population | 2 | 26.349 | 13.174 | 0.105 | 1.037% | 0.001 |
| Within populations | 145 | 1453.483 | 10.024 | 10.024 | 98.963% | 0.001 |
|  |  |  |  |  |  |  |
| Type 4: QD vs DL/ZH/GE/T | | | | | | |
| Among regions | 1 | 12.754 | 12.754 | 0.016 | 0.162% | 0.256 |
| Among population | 3 | 35.907 | 11.969 | 0.065 | 0.642% | 0.002 |
| Within populations | 145 | 1453.483 | 10.024 | 10.024 | 99.197% | 0.004 |
