# Supplementary material for: High migratory propensity constitutes a single stock of an exploited cutlassfish species in the Northwest Pacific: A microsatellite approach
Source: PLoS One. 2022 Mar 17;17(3):e0265548. doi: 10.1371/journal.pone.0265548 (PMC8929604; doi:10.1371/journal.pone.0265548)
Supplement: S8 Table — Nm = Number of migrants. Location 1 = DL, 2 = QD, 3 = ZH, 4 = GE, and 5 = T. (DOCX) [file pone.0265548.s010.docx]

S8 Table. The number of migrants per generation among locations. N_m_ = Number of migrants. Location 1 = DL, 2 = QD, 3 = ZH, 4 = GE, and 5 = T.

| Migrate | N_m_ |
| --- | --- |
| M1 to 2 | 18464.53 |
| M2 to 1 | 17301.54 |
| M1 to 3 | 16039.18 |
| M3 to 1 | 18642.04 |
| M1 to 4 | 19076.32 |
| M4 to 1 | 18319.18 |
| M1 to 5 | 15739.67 |
| M5 to 1 | 17872.33 |
| M2 to 3 | 17536.73 |
| M3 to 2 | 17958.68 |
| M2 to 4 | 19241.48 |
| M4 to 2 | 19656.04 |
| M2 to 5 | 17663.74 |
| M5 to 2 | 18358.79 |
| M3 to 4 | 17558.99 |
| M4 to 3 | 16684.66 |
| M3 to 5 | 16628.01 |
| M5 to 3 | 15889.64 |
| M4 to 5 | 16326.78 |
| M5 to 4 | 16519.21 |
