# Supplementary material for: High migratory propensity constitutes a single stock of an exploited cutlassfish species in the Northwest Pacific: A microsatellite approach
Source: PLoS One. 2022 Mar 17;17(3):e0265548. doi: 10.1371/journal.pone.0265548 (PMC8929604; doi:10.1371/journal.pone.0265548)
Supplement: S1 Raw images — (PDF) [file pone.0265548.s011.pdf]

S2 Figure_raw images

TJ-2

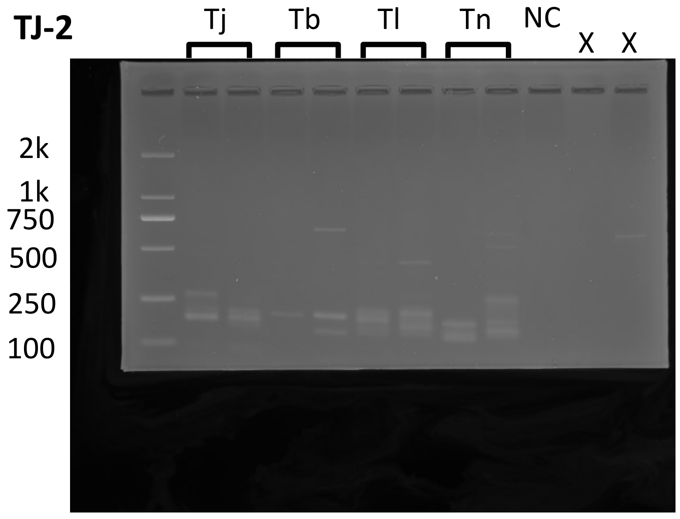

Canon PowerShot G16
2021-07-23 19:15:14
3.2 sec, f/2.8,
ISO 100, 0 EV

TJ-7

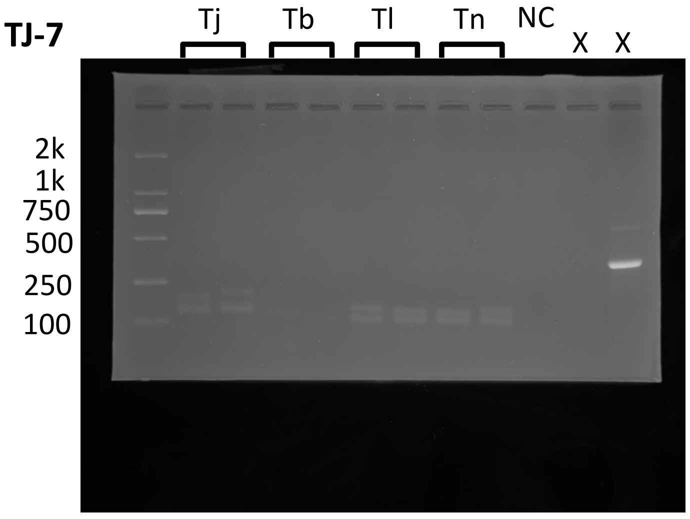

Canon PowerShot G16
2021-07-23 17:32:39
3.2 sec, f/2.8,
ISO 100, 0 EV

TJ-8

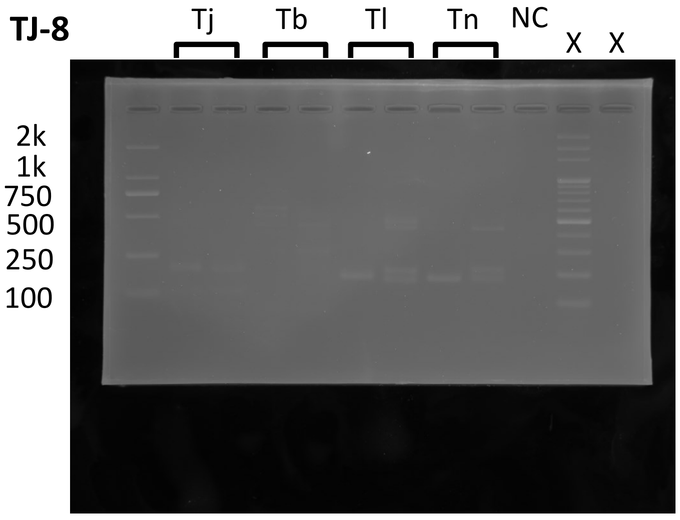

Canon PowerShot G16
2021-09-17 19:25:21
3.2 sec, f/2.8,
ISO 100, -1 EV

TJ-9

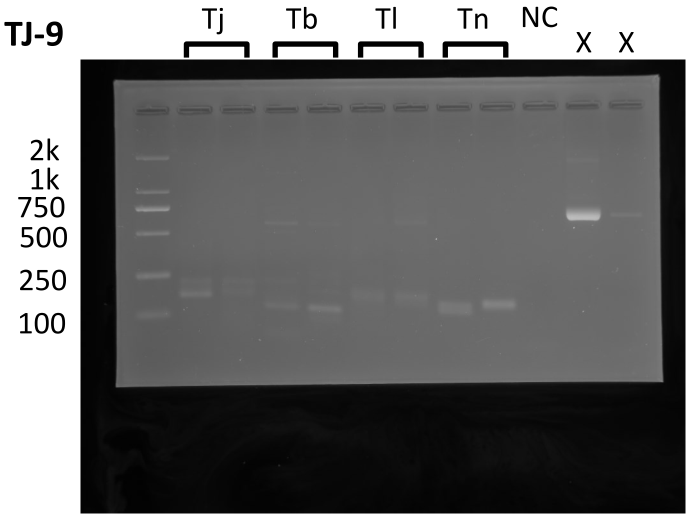

Canon PowerShot G16
2021-07-27 15:37:17
3.2 sec, f/2.8,
ISO 100, 0 EV

TJ-10

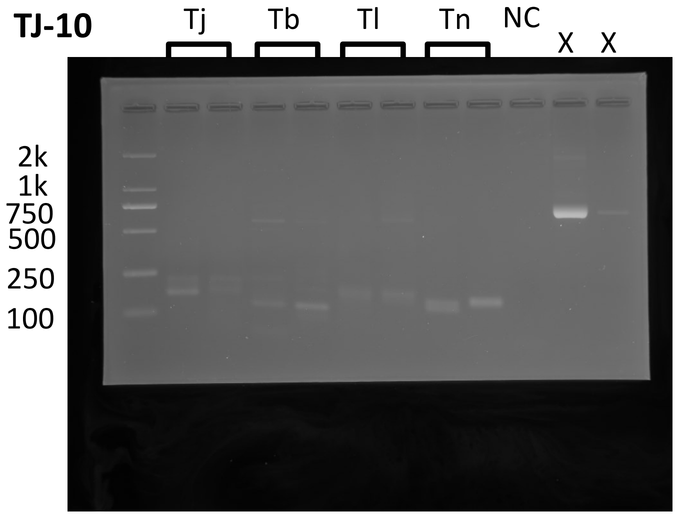

Canon PowerShot G16
2021-07-27 15:48:36
3.2 sec, f/2.8,
ISO 100, 0 EV

TJ-14

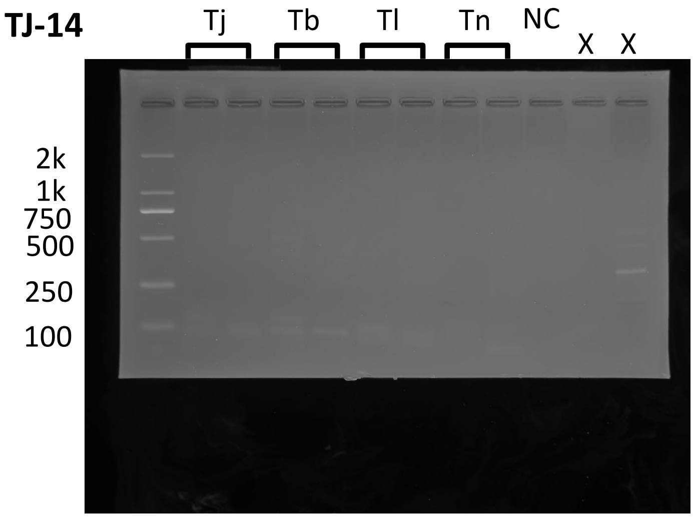

Canon PowerShot G16
2021-07-23 17:39:53
3.2 sec, f/2.8,
ISO 100, 0 EV

TJ-17

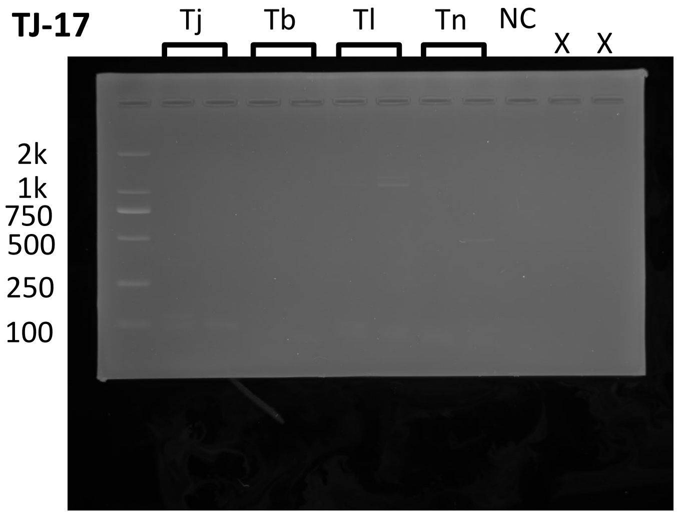

Canon PowerShot G16
2021-07-27 17:18:41
3.2 sec, f/2.8,
ISO 100, 0 EV

TJ-18

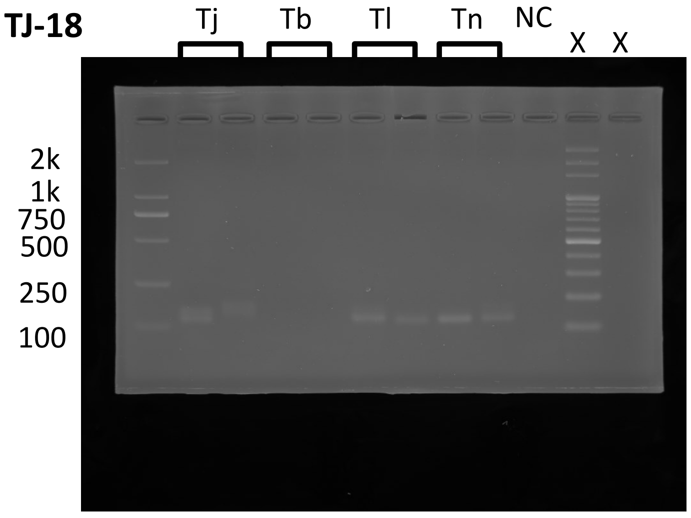

Canon PowerShot G16
2021-09-16 13:19:15
3.2 sec, f/2.8,
ISO 100, -1 EV

TJ-20

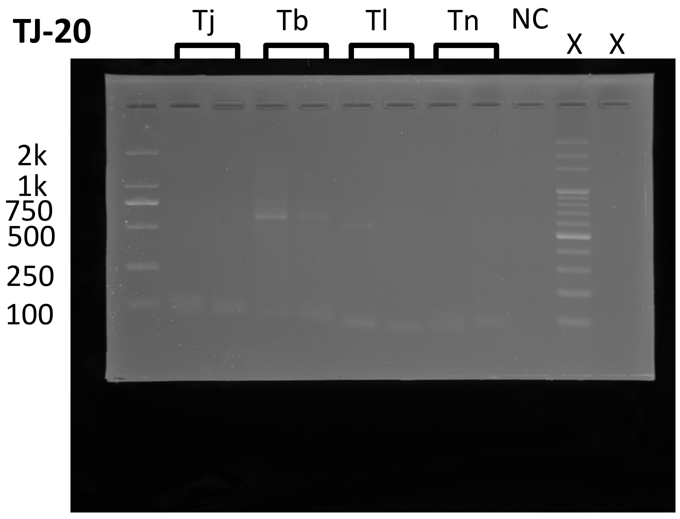

Canon PowerShot G16
2021-09-10 17:16:41
3.2 sec, f/2.8,
ISO 100, -1 EV

TJ-21

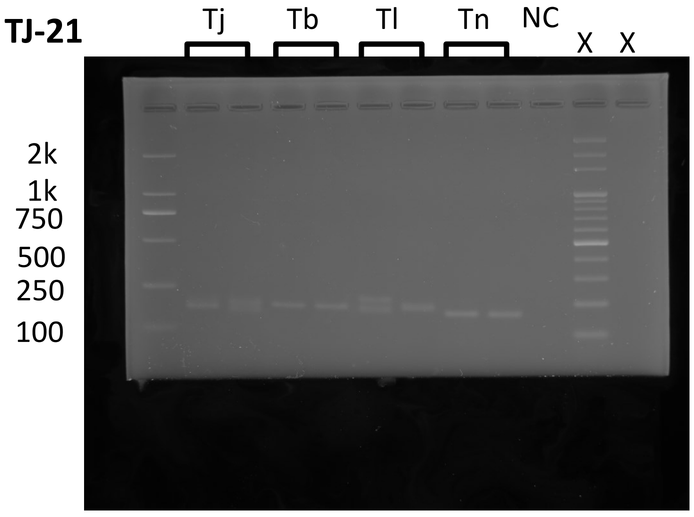

Canon PowerShot G16
2021-09-10 17:19:29
3.2 sec, f/2.8,
ISO 100, -1 EV

UV LightBox: 254nm (UV2020-2, TOP BIO CO.)
Gel documentation (DGIS-11c, TOP BIO CO.) with camera (Canon PowerShot G16)
